# Weed control efficiency and yield response of peanut (*Arachis hypogaea* L.) to different herbicides

**DOI:** 10.1038/s41598-026-42773-9

**Published:** 2026-04-29

**Authors:** Ramazan Gürbüz, Ramazan Taşkın, Harun Alptekin

**Affiliations:** 1 Department of Plant Protection, Faculty of Agriculture, Igdir University, 76000 Igdir, Türkiye; 2 Department of Animal Science, Faculty of Agriculture, Igdir University, 76000 Igdir, Türkiye

**Keywords:** Peanut, Weed management, Herbicides, Yield losses, Weed control efficiency, Ecology, Ecology, Environmental sciences, Plant sciences

## Abstract

Weeds are a major constraint to peanut (*Arachis hypogaea* L.) production, causing substantial yield and quality losses. This study was conducted during the 2020 and 2021 growing seasons to evaluate the effects of different pre- and post-emergence herbicides on weeds and peanut yield under field conditions. The experiment was arranged in a randomized complete block design with four replications. Treatments included one non-selective burndown herbicide (glyphosate), two pre-emergence herbicides (pendimethalin and dimethanamid p), four post-emergence herbicides (bentazon, quizalofop-p-ethyl, imazamox, clethodim), applied alone or in selected sequences. Weed control varied significantly with herbicides type, weed species, and evaluation time. Control levels increased markedly at 21 and 35 days after treatments (DAT) but declined by 105 DAT, indicating reduced residual activityand late weed emergence. The post-emergence combinations of bentazon + quizalofop-P-ethyl and bentazon + imazamox provided the highest weed control (88.2% and 88.9%, respectively) and significantly reduced weed dry biomass. Weed interference reduced peanut yield by approximately 60–70% copared with the weed-free control. All effective herbicide treatments significantly reduced weed biomass and prevent yield loss, leading to substiantially higher than the weedy control. Overall, the findings indicate that appropriate herbicide selection and optimized application timing play a critical role in achieving effective weed control and minimizing yield losses in peanut production.

## Introduction

Peanut (*Arachis hypogaea* L.) is an important oil plant in the world, belonging to the Fabaceae family^1,2^. Peanut is a valuable food source for humans and animals, is rich in oil, protein, carbohydrates, vitamins and minerals (Arıoğlu 2007)^3–5^. Peanut also stands out as a notable oil crop with its ability to fix nitrogen in the soil^6,7^. Peanut seeds contain 42–52% oil and 25–32% protein, and are especially evaluated in the production of peanut oil, snacks and peanut butter^5,8^. Additionally, peanut consumption has a positive effect on human health^3^.

Peanut has spread from South America to other parts of the world^9,10^. A total of 54.27 million tons were produced in 30.9 million hectares of land worldwide in 2023. 61% of this production was provided by Asia and 30% by Africa. In the same year, the countries that produced the most peanuts were China (19.2 million tons), India (10.2 million tons) and Nigeria (4.3 million tons), respectively^11^. In Turkey, peanut production has increased in recent years, and as of 2023, 185,137 tons were produced in 460,098 hectares^12^.

The rapid increase in the world population is increasing the demand for agricultural products and global agricultural production needs to be doubled by 2050^13,14^. Instead of opening new agricultural lands to increase yields, sustainable methods should be used^15,16^. However, there are various factors that negatively affect productivity in agricultural areas. One of the most important of these is weeds^17,18^.

Significant yield and quality losses are also observed in peanuts, which are of vital importance for global oil production^2^, due to weeds^19–30^. The peanut crop is highly susceptible to weed infestation due to its slow growth up to 40 days in the initial stages, short plant height and underground pod bearing habit^29,31^. Many weed species are a major constraint in peanut cultivation areas, causing yield losses ranging from 15 to 75%, depending on weed species composition, weed density, and environmental conditions^2,20,31,32^. In addition, weeds are preferred hosts of various insect pests and vectors of many important organisms that cause diseases in peanut^2,29^. Weeds also affect peanut through the production of harmful allelochemicals.

The intensity and duration of weed competition are critical determinants of yield loss in peanut prouction^33^. Accordingly effective weed control is a critical production factor in peanut cultivation and is essential for achieving acceptable yield and quality^31^. Weed control is therefore critical in peanut cultivation to achieve acceptable yield and quality. Although weed control can be achieved through manual weeding, this is generally expensive, time-consuming, laborious and impractical in under modern production conditions^23,34^. Consequently, herbicides are extensively used for weed control in peanut croping systems^19,20,22,24,25,27,35,36^. Following their widespread adoption after the Second World War^37^, chemical weed control has become one of the most commonly applied weed management strategies worldwide^11^. Despite the availability of various herbicides, no single product can provide season-long weed control in peanut because of differences in weed communities, limited residual activity, application timing constraints, and crop rotation considerations^20^. Therefore, effective weed management in peanut usually requires herbicide mixtures and/or sequential application of preplant, pre-emergence, and/or post-emergence herbicides^19,22,25,27^. However, the effectiveness of herbicides can vary significantly between years and locations depending on differences in environmental conditions and weed emergence dynamics^20^. This highlights the need for region-specific assessments. Accordingly, the aim of this study is to determine the weed control effectiveness and yield effects of selected herbicide programs in peanuts under local ecological conditions of Kozan (Adana Province), Turkey, in order to develop effective strategies to minimize weed-induced yield losses, and to contribute to sustainable peanut production.

## Material and method

### General characteristics of the experimental area

Two field trials were conducted in the Çukurköprü district, which is located 17 km from Kozan in the Adana province of Turkey, during the 2020–21 growing seasons, in order to investigate the impact of various herbicides on weed growth and peanut yield. The soil characteristics of the experimental area where the study was carried out are given in Table 1 and Table 2, and the climate data for the months of the study, 2020 and 2021, and LTA (long-term average) are given.

**Table 1:** Soil characteristics in the experimental area.

Soil properties	2020	2021
Profile depth (cm)	0–30	0–30
Soil texture class	clay-loam	clay-loam
Lime CaCO₃ (%)	27,98	26,41
Electrical conductivity (dS m⁻^1^)	0,02	0,015
pH*	7,88	7,75
Phosphorus (P₂O₅, mg kg⁻^1^)	4,22	3,90
Potassium (K₂O, mg kg⁻^1^)	365,3	380,1
Organic matter (%)	2,1	2,3

*Soil pH was measured in a 1:2.5 soil:water suspension.

**Table 2:** Monthly precipitation and mean temperature during the 2020 and 2021 growing seasons and long-term averages (LTA)^38^.

Months	Precipitation (mm)			Temperature (°C)		
	**2020**	**2021**	LTA*	**2020**	**2021**	LTA*
April	14,2	15,5	6,7	15,5	16,5	17,3
May	5,3	4,6	23,4	23,6	22,6	21,9
June	4,1	3,8	16,1	25,7	26,1	25,4
July	3,2	3,1	10,4	27,2	27,5	27,8
August	2,3	2,1	9,7	29,4	28,6	28,5
September	2,5	2,0	19,1	26,3	25,3	26,4

Meteorological Service (MS)^38^﻿. Temperature values represent monthly means. *LTA: Long-Term Average.

### Plant seed used, herbicides and setting up the experiment

The cultivar NC-7, widely grown under Çukurova conditions, was used as the plant material. One non-selective burndown herbicide (glyphosate), two pre-emergence (pendimethalin and dimethanamid-P) and four post-emergence (bentazon, quizalofop-p-ethyl, imazamox, clethodim) herbicides and their combinations were used in the experiment. The general characteristics of the herbicides used in the study are given in Table 3.

**Table 3:** General characteristics of the herbicides used in the study.

Code	Active ingredient	Formulation	Application rate	Application time
D	dimethenamid-P	EC	1500 mL ha^-1^ (1080 g a.i. ha⁻^1^)	Pre-emergence (immediately after planting)
P	pendimethalin	CS	3000 mL ha^-1^ (1350 g a.i. ha⁻^1^)	Pre-emergence (immediately after planting)
C	clethodim	EC	1000 mL ha^-1^ (240 g a.i. ha⁻^1^)	Post-emergence (21 days after planting)
BI	bentazon + Imazamox	SL	1800 mL ha⁻^1^ (864 g + 40.3 g a.i. ha⁻^1^)	Post-emergence (21 days after planting)
G	glyphosate isopropylamine salt	SL	3000 ml ha^-1^ (1440 g a.i. ha⁻^1^)	Burndown before crop emergence
Q	quizalofop-p-ethyl	EC	1250 mL ha^-1^ (62.5 g a.i. ha⁻^1^)	Post-emergence (21 days after planting)
B	bentazon	SL	2500 mL ha^-1^ (1200 g a.i. ha⁻^1^)	Post-emergence (21 days after planting)

EC: Emulsifiable Concentrate, CS: Capsule Suspension, SL – Soluble Liquid.

Since peanut was cultivated as the first crop, primary soil tillage was conducted using a moldboard plough in October following the harvest of the pre-plant in autumn and allowed to settle. After the rains, it was ploughed in reverse with a bottom trencher in January. After mixing superficially with a disc harrow in spring, the soil was left to stand for 1 day. Before planting, 300 kg ha⁻^1^ of diammonium phosphate (DAP) fertilizer was applied using a fertilizer spreader. Prior to planting, the seeds were treated with with azoxystrobin (75 g L⁻^1^), metalaxyl-M (37.5 g L⁻^1^), and fludioxonil (12.5 g L⁻^1^) against fungal diseases; dinotefuran against root collar pests (*Aspergillus niger* and *Cercospora* spp.); and imidacloprid (600 g kg⁻^1^) against wireworms (*Agriotes* spp.), following label recommendations. Peanut planting was conducted on 28 April 2020 and 2 May 2021 using a pneumatic seeder at a depth of 4–5 cm, with an intra-row spacing of 8.5 cm and a inter-row spacing of 70 cm. The planting norm was calculated as 16 plants m⁻^2^, corresponding to a seed rate of 144 kg ha⁻^1^. Irrigation was applied using a sprinkler system. Due to peanuts’ relatively low water requirements during the early growth stages, the first sprinkler irrigation was delayed until adequate vegetative development had occurred and visible symptoms of water stress had appeared. The first irrigation was applied at the early flowering stage, once the plants had developed an established root system. Owing to dry climatic conditions, a total of six sprinkler irrigations were applied throughout the growing season until harvest. The cumulative seasonal irrigation depth was 420 mm. During the growing period, total natural precipitation amounted to 31.6 mm in 2020 and 31.1 mm in 2021 (Table 2). Thus, the total water input (irrigation + rainfall) was 451.6 mm in 2020 and 451.1 mm in 2021. After planting, fertilization was done with the first sprinkler irrigation with 200 kg ha⁻^1^ urea (%46 N). At the fruiting stage, 150 kg ha⁻^1^ ammonium sulfate was applied, followed by 100 kg ha⁻^1^ calcium ammonium nitrate (26% N) with the fifth irrigation. In addition, 20 L ha⁻^1^ humic acid and 20 L ha⁻^1^ sulfur (80% sulfur) were applied through irrigation system. The trial was established according to a randomized complete block design with seven treatments consisting of pendimethalin, dimethenamid-P + glyphosate, clethodim, bentazon + quizalofop-P-ethyl, bentazon + imazamox, a weedy control, and a weed-free control and four replications. Plot size was 3.5 × 3.0 m (10.5 m^2^), and the total experimental area was 459 m^2^. To prevent spray drift, a 1-m buffer zone was maintained between plots, and the inter-plot alleys were mechanically hoed to avoid interference between the different treatments. Glyphosate was applied only in the relevant treatment plots as a non-selective burndown treatment one day after planting, before the emergence of peanuts, to control perennial weeds that had survived tillage. Although the field was conventionally tilled, perennial species such as *Cyperus rotundus* and *Sorghum halepense* can survive soil disturbance through underground propagules, necessitating a pre-emergence burndown application. Dimethenamid-P and pendimethalin were applied immediately after planting((0 days after planting; 28 April 2020 and 2 May 2021) as pre-emergence treatments, while clethodim, bentazon + imazamox and bentazon + quizalofop-P-ethyl were applied 21 days after planting (21 DAP) as post-emergence treatments. Accordingly, pre-emergence assessments refer exclusively to dimethenamid-P and pendimethalin treatments, whereas post-emergence assessments correspond to clethodim, bentazon + imazamox, and bentazon + quizalofop-P-ethyl applications.

All herbicide treatments were applied using a backpack sprayer equipped with flat-fan nozzles, delivering a spray volume of 300 L ha⁻^1^ at a constant pressure of 300 kPa. Applications were conducted under calm weather conditions (wind speed < 3 m s⁻^1^, temperature 20–28 °C, relative humidity 50–65%). No additional adjuvants were used unless specified on the product label.

### Determination of weed species and densities in the trial area

In the study, in order to determine the weed species and densities in the trial area, a 1 m^2^ frame was used in the trial area and the weeds in the frame were counted by where quadrats were placed. The evaluation was made based on the arithmetic mean in determining the density of weeds. The density of individual species was calculated by dividing the total number of plants per m^2^ (plants m⁻^2^) by the number of frames thrown^39^.

$$\text{Density }(\mathrm{plant}/{\mathrm{m}}^{2})=\frac{\mathrm{B}}{\mathrm{M}}$$

B; Total number of individuals in the sample taken (plant).

M; Number of frames thrown (m^2^).

In addition, according to Üstüner and Güncan^40^, weed density in the experimental area was classified using a standard density scale. Species with more than 10 plants m⁻^2^ were considered high density, those with 1–10 plants m⁻^2^ as dense, those with 0.1–1 plants m⁻^2^ as moderate density, those with 0.01–0.1 plants m⁻^2^ as low density, and those with fewer than 0.01 plants m⁻^2^ as rare.

### Determination of the effects of herbicides on weed density and species in the study

Pre-emergence herbicides were applied immediately after sowing, while post-emergence herbicides were applied 21 days afterwards. Weed evaluations were not conducted separately for each treatment based on its application time. Instead, all weed assessments were performed on the same field observation dates for all treatments. These evaluation dates, which corresponded to 7, 21, 35 and 105 days after the post-emergence application (DAT), represented the early, mid-season and pre-harvest periods. This approach ensured that the performance of the herbicides was compared at identical stages of crop and weed development, thereby enabling clear interpretation of the effects of the treatments within the crop cycle.

In the study, a total of four evaluations were made at certain intervals throughout the trial period in order to determine the effects of herbicides on weed density and species^41^. In the evaluations, weed density and weed species were considered separately and changes in both parameters after herbicide applications were assessed. These changes were expressed as reductions in weed density. Herbicide-treated plots were compared with untreated control plots, and weed control (%) was calculated as the proportional reduction in weed density relative to the control, following a relative efficacy correction approach commonly used in applied weed science^41^. Weed control (%) was calculated as:

$$Weed \:control \left(\%\right)=\left(\frac{Weed\: density\: in\: control-Weed\: density\: in\: treatment}{Weed\: density\: in\: control}\right)\times 100$$

**Fig. 1:**
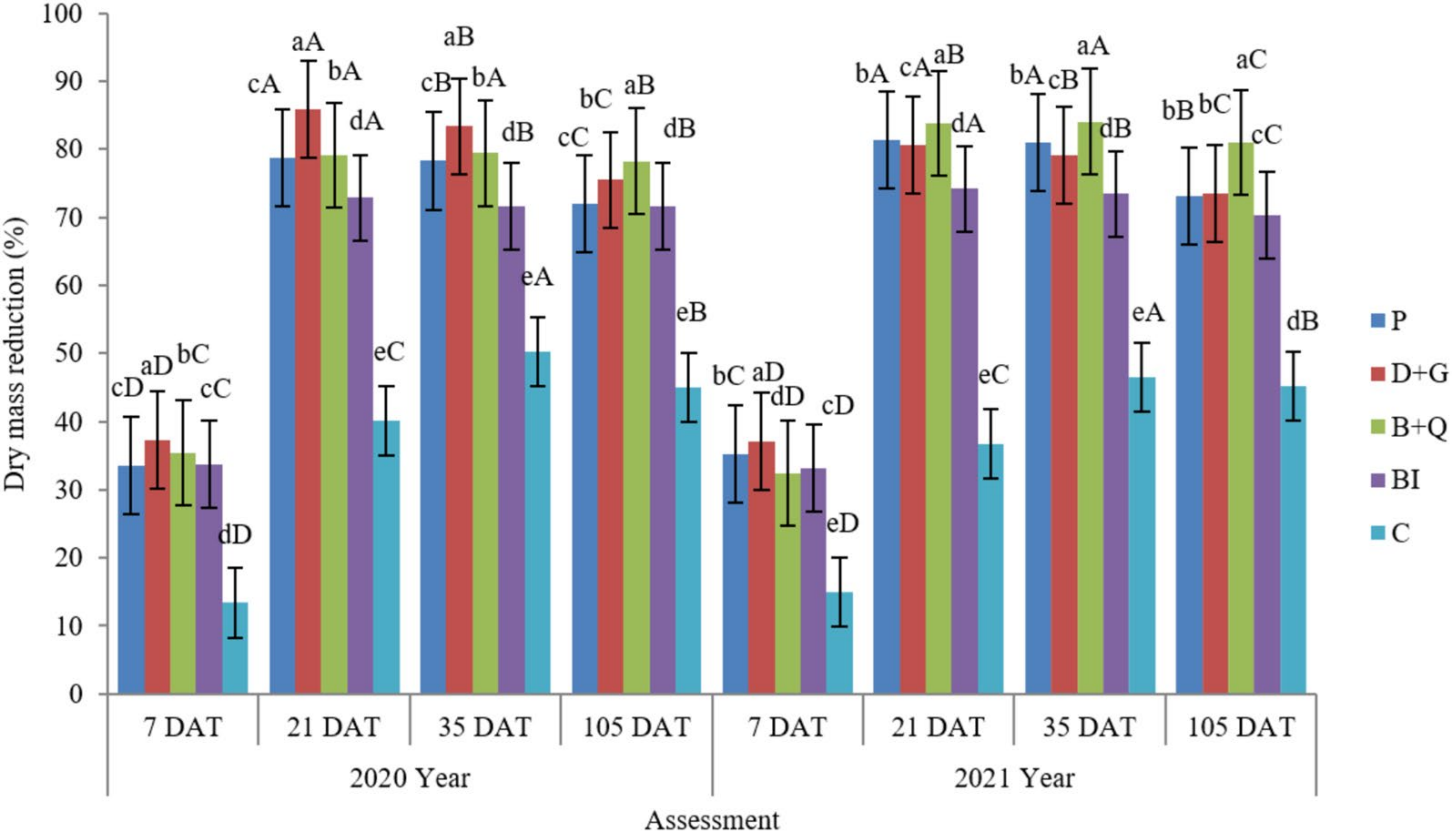
Effects of herbicides on weed density. DAT: Days After Treatment: D: Dimethenamid-P, P: Pendimethalin, C: Clethodim, BI: Bentazon + Imazamox, G: Glyphosate isopropylamine salt, Q: Quizalofop-p-ethyl, and B: Bentazon. Lowercase letters indicate statistical groupings among different treatments, while uppercase letters represent statistical differences among evaluations within each treatment. Means followed by the same letter are not significantly different at the 0.05 probability level.

**Fig. 2:**
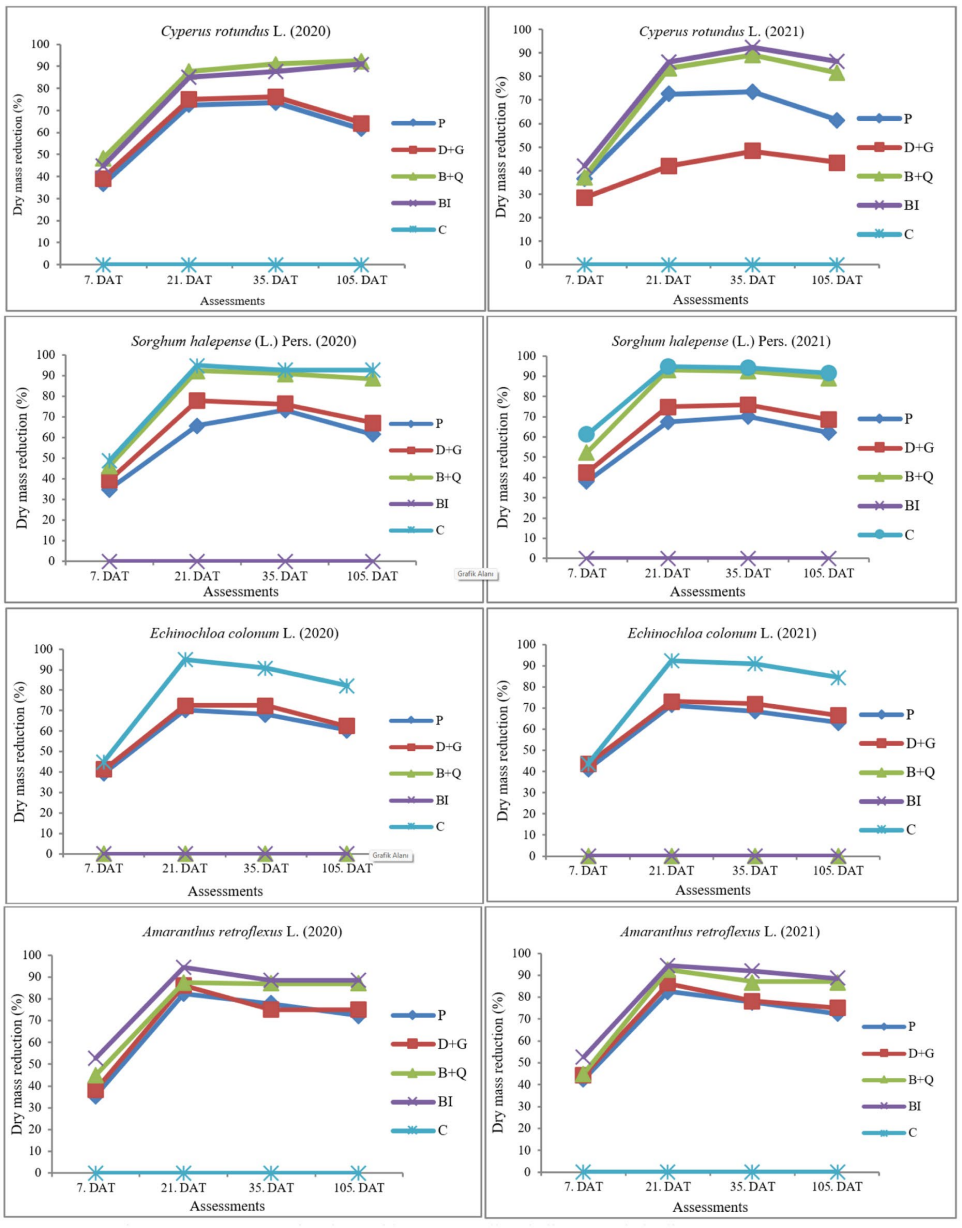
Effects of herbicides on weed species (*Cyperus rotundus, Sorghum halepense Echinochloa colonum, Amaranthus retroflexus*). DAT: Days After Treatment. D: Dimethenamid-P, P: Pendimethalin, C: Clethodim, BI: Bentazon + Imazamox, G: Glyphosate isopropylamine salt, Q: Quizalofop-p-ethyl, and B: Bentazon.

**Fig. 3:**
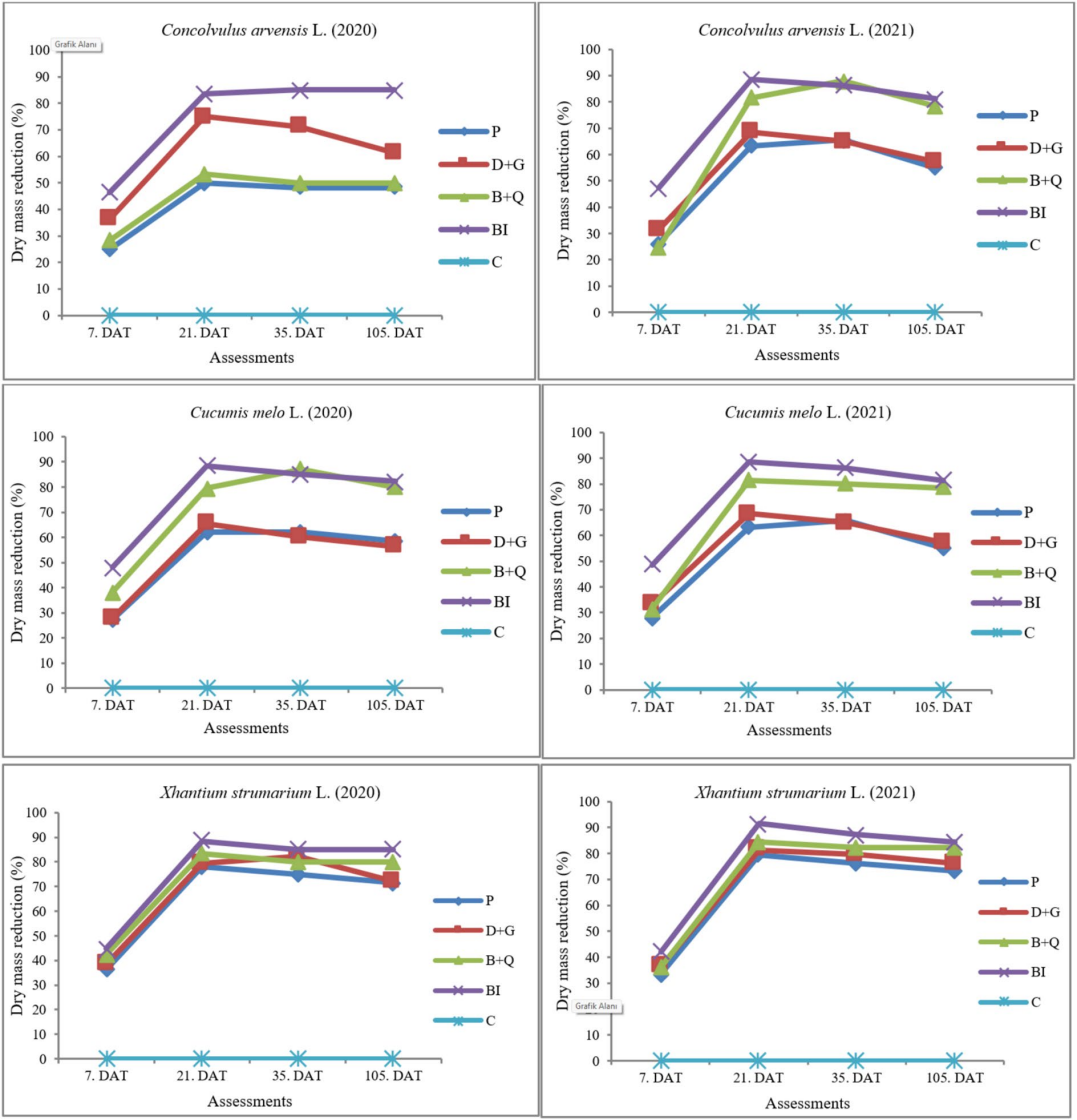
Effects of herbicides on weed species (*Convolvulus arvensis, Cucumis melo, Xanthium strumarium)*. DAT: Days After Treatment. D: Dimethenamid-P, P: Pendimethalin, C: Clethodim, BI: Bentazon + Imazamox, G: Glyphosate isopropylamine salt, Q: Quizalofop-p-ethyl, and B: Bentazon.

This calculation is mathematically equivalent to the correction originally described by Abbott^42^ and is adapted here to standardize weed control assessments relative to untreated plots. The values presented in Fig. 1 were calculated as the percentage reduction in weed density relative to the weedy control. This was achieved by comparing the weed density recorded in each treated plot with that in the corresponding untreated control plot, using the relative efficacy formula. Treatment means were then calculated from the percentage values recorded at plot level. The resulting weed control (%) values were used as the effectiveness metric for all herbicide evaluations presented in Figs. 1–3 and Tables 7–8.

### Effects of herbicides on weed fresh and dry biomass

Weed biomass was sampled at the final evaluation (105 days after treatment; 105 DAT). Within each plot, all weeds were harvested at soil level from a 1 m^2^ quadrat located in the center of the plot to avoid border effects. To minimize moisture loss, the harvested weeds were weighed immediately in the field using a portable digital balance. The samples were then placed in labelled paper bags and transported to the Herbology Laboratory at Iğdır University’s Faculty of Agriculture. The weed samples were then oven-dried at 70 °C for 24 h until they reached a constant weight, after which their dry weights were recorded. The herbicide effect on weed biomass was calculated as a percentage reduction relative to the weedy control plots.

### Effects of herbicides on peanut yield and yield components

Peanut harvesting was carried out on 25 September 2021 and 18 September 2022 at physiological maturity. Within each plot, a 1 m^2^ area was hand-harvested from the central rows to determine peanut yield and yield components. After harvesting, the plants were air-dried and the pods separated. The yield was then recorded. Peanut yield and selected yield components, including pod yield, thousand-kernel weight, number of kernels per 100 g, and kernel yield per unit area, were evaluated following Canavar and Kaynak^43^ and Williams and Drexler^44^.

### Data analysis

As a result of four different counts made in the study, the weed control (%) rates of herbicides, weeds fresh and dry weights, and peanut yield and yield elements were evaluated. Weed density and biomass data were calculated as means across weed species Data from both experimental years were first tested together. Weed fresh and dry weights were analyzed using a two-way analysis of variance (ANOVA) with year and herbicide treatment as fixed factors. It was determined that herbicide applications had statistically significant effects on both the fresh and dry weights of weeds (p < 0.001). The year × application interaction was found to be significant for the fresh weight of weeds (p = 0.014), indicating that the effectiveness of the applications varied over the years. In contrast, the year × application interaction was not statistically significant for the dry weight of weeds (p = 0.823). Peanut yield and yield components were also analyzed using a two-way ANOVA with year and herbicide treatment as fixed factors. Herbicide treatment had a highly significant effect on all yield parameters (p < 0.001), whereas the main effect of year was not significant. The year × treatment interaction was significant only for yield (p = 0.006), indicating that treatment effects on yield differed between years.

Mean comparisons were performed using Duncan’s multiple range test at p < 0.05 (SPSS 20). Additionally, correlation analysis (JASP) was used to determine relationships among variables after data transformation/normalisation. Heatmap and hierarchical clustering (SRplot) were conducted to visualise data density and group applications according to similarity. Network graph analysis and principal component analysis (PCA) were performed using PAST software to visualise relationships among the variables, reduce the multivariate data to a lower dimension and identify the most influential variables.

## Results and discussion

### Weed species detected in the experimental area and their densities

Weed species detected in both years, their general characteristics and densities are given in Table 4. Weed density values represent mean counts recorded prior to herbicide application and reflect the baseline weed flora for each growing season.

**Table 4:** Weed species detected and their life cycle and density characteristics.

Family	Weed Name	LC	Density (plants m^-2^), Intensity scale	
			**2020**	**2021**
**Narrow-leaf**				
Poaceae	*Echinochloa colonum* (L.) Link	A	12.50H	10.25 H
	*Sorghum halepense* (L.) Pers	P	15.34 H	14.50 H
Cyperaceae	*Cyperus rotundus* L	P	23.25 H	20.50 H
**Broad-leaf**				
Amaranthaceae	*Amaranthus retroflexus* L	A	5.33 M	4.00 M
Asteraceae	*Xanthium strumarium* L	A	5.70 M	3.50 M
Convolvulaceae	*Convolvulus arvensis* L	P	9.52 M	7.50 M
Cucurbitaceae	*Cucumis melo* L. subsp. Agrestis (Naudin.)	A	8.72 M	7.50 M

LC; Life Cycle, A; Annual, P; perennial, H; High density–density = 10 + plants m⁻^2^, M; Dense-density = 1–10 plants m⁻^2^.

In the study, seven weed species were identified and they belong to six different families. Two of these families are narrow-leaved and four are broad-leaved families. *C. rotundus* showed the highest density (2020: 23.25 plants m⁻^2^, 2021: 20.50 plants m⁻^2^). *S. halepense* and *E. colonum* were also among the important weeds. In addition, three weed species were determined as high density (density =  + 10 plants m⁻^2^) and four weed species were determined as dense (density = 1–10 plants m⁻^2^) in both years. The high density of *C. rotundus* confirms the prevalence of *Cyperus* species reported in previous studies such as^20,45^. In present study, species such as *E. colona* and *S. halepense* were identified as major weed species in peanut fields. In peanut fields of southern Turkey, weed communities are characterized by high species richness and density, with Poaceae species forming a major component of the weed flora^46^. The high dominance of these species is consistent with findings reported by^47^ in peanut and other summer row-crop systems. Moreover, previous studies have indicated that the density of broad-leaved weed species has increased in peanut cultivation areas, resulting in greater management challenges^20,48^.

### Effect of herbicides on weeds

The herbicides used in the study showed different effects on the weed density. The effects of herbicides on the weed density are shown in Fig. 1.

In the study, significant differences were observed in the effectiveness of herbicides between 2020 and 2021 (Fig. 1.). The presented values represent means averaged over weed species. According to Fig. 1, bentazon + quizalofop-P-ethy generally exhibited high weed control, , particularly in the mid-season evaluations, whereas the lowest effectiveness was consistently observed with clethodim (2020: 45.0%; 2021: 45.2%). These results indicates that herbicide performance varied between years and evaluation times. While the effects were lower in the early and late periods such as seven DAT and 105 DAT, the effectiveness of herbicides increased significantly, especially at 21 DAT and 35 DAT (Fig. 1.). In all herbicides, a decrease in weed control (%) was observed in the last counts after 35 DAT. However, pendimethalin (P), dimethenamid-P + glyphosate (D + G), and bentazon + quizalofop-P-ethyl (B + Q) were more effective, maintaining higher and more stable weed control levels across the evaluation periods after 7 DAT (Fig. 1). In contrast, clethodim (C) showed persistently lower control efficacy, with values generally remaining between 40 and 50%. These results indicate that, beyond the initial assessment, herbicide treatments differed markedly in the stability of their weed control over time. It is of great importance for peanut producers to consider the correct timing when choosing the most suitable herbicide to ensure effective weed control. In addition, greater efficacy was observed when the herbicides used are applied by considering the weed species in the relevant area. The effects of herbicides on weed species are given in Fig. 2 and Fig. 3.

The effectiveness of the herbicides used in this study depended primarily on herbicide type, weed species and application timing. This reflects differences in the mode of action of the herbicides and the biology of the weeds. Dimethamid-P + glyphosate and bentazon + quizalofop-P-ethyl were the most consistently effective treatments overall, providing broad-spectrum control across multiple weed species and achieving the highest levels of efficacy. High levels of weed control using herbicide mixtures have previously been reported in peanuts and other summer crops, where combinations improve control by targeting both grass and broad-leaved weeds simultaneously^20,24^. Evident species-specific responses were observed. For *Cyperus rotundus*, weed control ranged from 60 to 89%, with the highest efficacy being provided by bentazon + imazamox, while clethodim was ineffective in both years. This is consistent with previous studies showing that herbicides inhibiting ALS and PSII are effective against sedge species^45,48^. By contrast, clethodim, an acetyl-CoA carboxylase (ACCase) inhibitor, exhibited minimal activity against broad-leaved weeds such as *Amaranthus retroflexus*, *Xanthium strumarium, Cucumis melo* and *Convolvulus arvensis.* This is anticipated given its grass-selective mode of action and has been extensively documented in the weed science literature^49,50^. Grass weeds such as *Sorghum halepense* and *Echinochloa colona* could be effectively controlled using grass-active herbicides or mixtures containing quizalofop-P-ethyl or clethodim, often with an efficacy level of over 80–90%. These results support earlier reports that ACCase inhibitors and their combinations are effective in controlling annual and perennial grasses post-emergence in peanut cultivation^22,36,51^. However, bentazon + imazamox performed poorly on several grass species, highlighting the importance of selecting herbicides according to weed functional groups.

The timing of evaluation after application played a critical role in the performance of herbicides. Weed control values were generally lower at 7 days after treatment (DAT) and increased markedly at 21 and 35 DAT. Because weed control (%) was calculated based on reductions in weed density relative to the untreated control rather than immediate visual injury, later assessments represent cumulative herbicide effectiveness rather than increasing herbicide activity. This reflects the time required for herbicide absorption, translocation and cumulative weed mortality, particularly for systemic post-emergence herbicides. The decline in efficacy observed at 105 DAT is likely to be associated with reduced residual activity and late-emerging weeds; this pattern is commonly reported for both soil-applied and post-emergence herbicides^20,32^. These findings suggest that effective weed management in peanut cultivation requires not only appropriate herbicide selection and knowledge of species-specific susceptibility, but also optimised application timing. The effects of herbicide treatments on weed fresh and dry weights are presented in Table 5.

**Table 5:** Effects of herbicides on weed fresh and dry weights (gm⁻^2^).

Weeds fresh weight							
**Treatments**	**2020**			**2021**		**Mean**	
	Weed fresh weight(g m^-2^) ± SE		Effect (%)	Weed fresh weight (g m^-2^) ± SE	Effect (%)	Weed fresh weight (g m^-2^) ± SE	Effect (%)
Weedy control	3582,5 ± 177,22^a^		0,00	3187,50 ± 99,78^a^	0,00	3385,00a ± 78,78^a^	0,00
P	513,75 ± 34,54^b^		85,66	528,75 ± 29,03^b^	83,41	521,25 ± 29,12^b^	84,60
D + G	482,5 ± 20,86b		86,53	501,25 ± 21,44^bcc^	84,27	491,88 ± 20,92^b^	85,47
B + Q	351,25 ± 22,30b		90,20	360,00 ± 16,70^ cd^	88,71	355,63 ± 18,88^c^	89,49
BI	331,25 ± 10,87b		90,75	333,75 ± 14,77^d^	89,53	332,50 ± 12,62^c^	90,18
C	391,25 ± 42,14b		89,08	373,75 ± 29,88^ cd^	88,27	382,50 ± 35,69^c^	88,70
Mean	942,08 ± 76,34			882,83 ± 216,26		912,455 ± 187,26	
F	447,915			608,676		586,156	
*P value*	0,000			0,000		0,000	
**Weed dry weight**							
**Treatments**	**2020**			**2021**		**Mean**	
	Weed dry weight (g m^-2^) ± SE	Effect (%)		Weed dry weight (g m^-2^) ± SE	Effect (%)	Weed dry weight (g m^-2^) ± SE	Effect (%)
Weedy control	1188,75 ± 75,45^a^	0,00		1004,25 ± 42,00^a^	0,00	1096,50 ± 51,53^a^	0,00
P	198,75 ± 12,14^b^	83,28		187,50 ± 9,68^b^	81,33	193,13 ± 10,27^b^	82,39
D + G	187,50 ± 8,53^b^	84,23		186,25 ± 10,48^b^	81,45	186,88 ± 9,31^bc^	82,96
B + Q	125,00 ± 8,89^b^	89,48		133,75 ± 7,18^bc^	86,68	129,38 ± 7,59^bc^	88,20
BI	123,75 ± 4,26^b^	89,59		120,00 ± 4,56^c^	88,05	121,88 ± 4,37^c^	88,89
C	141,25 ± 9,65^b^	88,12		136,25 ± 9,86^bc^	86,43	138,75 ± 8,92^bc^	87,35
Mean	327,50 ± 81,35			315,41 ± 76,34	73,08	321,45 ± 78,64	74,87
F	175,972			284,227		265,786	
*P value*	0,000			0,000		0,000	
Source	df	Fresh weight			Dry weight		
		F		p-value	F	p-value	
Year	1	2.81		0.102	0.64	0.430	
Treatments	5	735.82		< 0.001	492.57	< 0.001	
Year × Treatment	5	3.36		0.014	0.43	0.823	
Error	36	–		–	–	–	

D: Dimethenamid-P, P: Pendimethalin, C: Clethodim, BI: Bentazon + Imazamox, G: Glyphosate isopropylamine salt, Q: Quizalofop-p-ethyl, and B: Bentazon. Means followed by the same letter are not significantly different at the 0.05 probability level.

Weed fresh and dry weights were analyzed using a two-way ANOVA with year and herbicide treatment as fixed factors. Herbicide treatments had a highly significant effect on both weed fresh and dry weights (p < 0.001). The year × treatment interaction was significant for weed fresh weight (p = 0.014), indicating that treatment performance varied between years, whereas no significant interaction was observed for weed dry weight (p = 0.823).

In both years, herbicide applications significantly reduced weed fresh and dry weights biomass. Compared to the weedy control group, herbicide applications significantly reduced the fresh weight of weeds. The highest effect was provided by bentazon + quizolofop-p-ethyl (B + Q) and bentazon + imazamox (BI) combinations. Clethodim (C) resulted in an 88.7% reduction in fresh weed biomass. Because biomass values were averaged across weed species, the apparent biomass reduction under clethodim mainly reflects suppression of grass weeds rather than broad-leaved species, for which clethodim is not expected to be effective. Similarly, herbicide treatments reduced weed dry biomass. Bentazon + quizalofop-P-ethyl and BI combinations showed the highest effectiveness by reducing dry weights by 88.2% and 88.9%, respectively.

Overall, bentazon + quizalofop-P-ethyl and bentazon + imazamox provided the most consistent biomass suppression under the conditions of this study. As a result, the herbicide applications effectively reduce the biomass of weeds, which provides a significant improvement in the growth of crop plants such as peanut. Previous studies report that weed control levels vary with herbicide mode of action and the dominant weed flora^25,52^. For example, it has been emphasized that some herbicides such as Propaquizafop are quite effective on reeds and grassy weeds, but their effectiveness on broadleaf weeds may vary. In our findings, the effectiveness of clethodim was lower (45%), which is consistent with the literature describing the effectiveness of different herbicides against different weed species^22,53^ also supports the effect of application timing on effectiveness^53^ also stated that herbicide applications made in the early period generally provide lower yields, but greater efficacy was observed with the right timing. All these findings show that the effectiveness of herbicides in weed control varies not only depending on the type of herbicide used, but also on the application time. Studies such as^27,54^ also reveal that herbicides can effectively reduce weed density and thus maintain yield potential when applied at the right time and appropriately. Therefore, it is critical for peanut producers to pay attention to herbicide selection and application timing to ensure effective weed control.

### Effect of herbicides on peanut yield and yield components

Herbicide treatments varied in their effects on peanut yield but significantly reduced yield loss caused by weed competition through effective weed control. The effects of herbicides on peanut yield and yield components are given in Table 6.

**Table 6:** Effects of herbicides on peanut yield and yield components.

2020									
Treatments		Number of Grains per 100 g		Thousand Kernel Weight (g)		Shelling percentage (%)		Grain Yield (kg ha^-1^)	
Weedy control		131,00 ± 5,11^a^		650,25 ± 8,15^d^		61,25 ± 1,04^c^		17,2 ± 0,9^c^	
Weed-free control		96,00 ± 2,48^d^		899,75 ± 13,91^a^		74,77 ± 0,49^a^		51,9 ± 0,9^a^	
P		107,75 ± 2,28^bc^		782,75 ± 13,23^bc^		71,85 ± 0,45^b^		42,7 ± 0,5^b^	
D + G		113,00 ± 1,58^b^		771,25 ± 8,86^c^		70,95 ± 0,75^b^		42,5 ± 0,5^b^	
B + Q		105,25 ± 2,39^bc^		797,00 ± 5,18^bc^		72,00 ± 0,30^b^		43,8 ± 0,8^b^	
BI		113,75 ± 1,88^b^		791,00 ± 10,83^bc^		72,22 ± 0,13^b^		43,0 ± 0,3^b^	
C		102,75 ± 1,97^ cd^		812,00 ± 4,93^b^		72,45 ± 0,36^b^		44,5 ± 0,7^b^	
Mean		109,92 ± 2,18		786,28 ± 13,50		70,77 ± 0,80		40,8 ± 19,6	
F		16,218		55,406		56,854		220,122	
*P value*		0,000*		0,000*		0,000*		0,000*	
**2021**									
Weedy control		127,75 ± 2,39^a^		609,50 ± 5,92^d^		61,45 ± 0,43^d^		15,7 ± 0,7^e^	
Weed-free control		94,50 ± 1,25^c^		893,50 ± 10,60^a^		74,60 ± 0,39^a^		53,2 ± 0,6^a^	
P		113,25 ± 2,13^b^		783,75 ± 20,09^bc^		71,40 ± 0,58^bc^		42,8 ± 0,5^ cd^	
D + G		110,50 ± 1,70^b^		796,00 ± 6,46^bc^		72,43 ± 0,35^bc^		43,1 ± 0,5^c^	
B + Q		109,00 ± 2,67^b^		774,75 ± 6,96^c^		71,58 ± 0,62^bc^		42,4 ± 0,5^ cd^	
BI		109,00 ± 1,25^b^		785,00 ± 9,97^bc^		71,07 ± 0,66^bc^		41,2 ± 0,4^d^	
C		102,25 ± 3,35^c^		807,50 ± 6,11^b^		73,00 ± 0,48^b^		47,8 ± 0,4^b^	
Mean		110,03 ± 1,92		778,85 ± 15,51		70,85 ± 0,78		40,8 ± 1,9	
F		19,923		64,627		68,927		384,305	
*P value*		0,000*		0,000*		0,000*		0,000*	
**Means**									
Weedy control		129,37 ± 3,08^a^		629,87 ± 4,19^d^		61,35 ± 0,65^c^		16,4 ± 0,7^d^	
Weed-free control		95,25 ± 1,66^d^		896,62 ± 10,76^a^		74,68 ± 0,13^a^		52,6 ± 0,3^a^	
P		110,50 ± 2,15^b^		783,25 ± 11,19^c^		71,62 ± 0,41^b^		42,8 ± 0,5^c^	
D + G		111,75 ± 1,56^b^		783,62 ± 3,71^c^		71,69 ± 0,51^b^		42,8 ± 0,5^c^	
B + Q		107,12 ± 2,11^bc^		785,87 ± 3,08^c^		71,79 ± 0,21^b^		43,1 ± 0,5^c^	
BI		111,37 ± 1,05^b^		788,00 ± 9,22^c^		71,64 ± 0,35^b^		42,1 ± 0,3^c^	
C		102,50 ± 2,49^c^		809,75 ± 2,93^b^		72,72 ± 0,31^b^		46,1 ± 0,5^b^	
Mean		109,97 ± 1,98		782,56 ± 14,23		70,81 ± 0,78		40,8 ± 1,9	
F		24,404		115,488		115,173		488,138	
*P value*		0,000*		0,000*		0,000*		0,000*	
Source	df	f	p* value*	f	p* value*	f	p* value*	f	p* value*
Year	1	0.006	0.937	1.850	0.181	0.071	0.791	0.015	0.903
Treatment	6	34.493	0.000	118.673	0.000	123.350	0.000	558.479	0.000
Year × Treatment	6	0.867	0.527	1.967	0.092	1.110	0.373	3.610	0.006
Error	42	–	–	–	–	–	–	–	–

D: Dimethenamid-P, P: Pendimethalin, C: Clethodim, BI: Bentazon + Imazamox, G: Glyphosate isopropylamine salt, Q: Quizalofop-p-ethyl, and B: Bentazon. Means followed by the same letter are not significantly different at the 0.05 probability level.

Peanut yield and yield components were analyzed using a two-way ANOVA with year and herbicide treatment as fixed factors. Herbicide treatment had a highly significant effect on all yield parameters (p < 0.001), whereas the main effect of year was not significant. The year × treatment interaction was significant only for yield (p = 0.006), indicating that treatment effects on yield varied between years.

Compared to the weed control, an increase in yield was observed in the plots where herbicides were applied. The yield in the weed-free control application was at the highest level in both years. However, herbicides also made a significant contribution and significant increases were obtained in terms of yield. In 2020 and 2021, herbicide applications provided an average increase in yield of 60% to 70%. While the bentazon + quizalofop-P-ethyl combination achieved the highest yield in both years, the clethodim application provided a significant yield increase in 2021. While Clethodim was not among the highest-performing treatments in terms of overall weed control efficacy, it did provide some of the highest yield values, particularly in the second year. This significant divergence suggests that the yield response was shaped not only by the overall weed suppression level but also by the selective control of the most competitive weed groups. Clethodim’s status as a grass-specific ACCase inhibitor and its strong suppression of Poaceae species may have been disproportionately effective in preventing yield loss. Grass weeds are often among the most aggressive competitors in peanut fields due to their rapid early development, high nutrient and water uptake capacity, and strong interference with canopy formation. Field observations during the trial period revealed a significant increase in the relative abundance of Poaceae species, particularly in the second year. This explains why selective grass control with clethodim resulted in significant yield conservation, despite the moderate overall weed suppression level. Similarly, selective control based on targeting dominant weed groups has been reported to provide a higher yield advantage in different summer crops compared to broader but less focused suppression of mixed weed communities^36^, (Jordan et al., 2013).

These results clearly demonstrate that effectively preventing yield loss depends not only on the magnitude of total weed reduction but also on targeting the species within the weed flora that most strongly limit yield. Herbicide applications that provided effective weed control significantly reduced peanut yield loss, with bentazon + quizalofop-P-ethyl, bentazon + imazamox, and clethodim showing the most effective results. These findings demonstrate the strong negative impact of weeds on peanut yield and the critical role of herbicides in alleviating this effect and improving productivity. These findings emphasize the importance of effective weed management strategies in peanut (*Arachis hypogaea* L.) production and are consistent with previous studies conducted in peanut systems by^25,47^. Herbicide applications provided similar yields when compared to weed-free controls^2^ stated that peanut production decreased by 5–15% due to weeds, and this loss reached up to 48.3% especially in areas with 20 weeds per square meter. These findings are consistent with the negative effects of weeds on yield observed in present study. Studies such as^55,27^ also emphasize the effect of herbicides in weed control. They state that herbicides such as Pendimethalin are especially effective in increasing peanut yield^19^ also stated that pre-emergence herbicide applications had a high effect on yield. Similarly^53,31^ studies show that weeds cause significant damage to peanuts and that these damages can be eliminated with herbicide applications.

### Multivariate analysis of parameters and applications

In addition to one-way variance analysis, the obtained mean values were subjected to a series of statistical analyses to visualize and correlate them. Since weed control (%), fresh and dry weights are critical issues considered in agricultural/non-agricultural areas, their relationships with other parameters were addressed. In this context, advanced analyses as correlation coefficient, heat map clustering, network graph analysis, hierarchical cluster analysis and principal component analysis were performed on the mean values of the variables in the study.

**Fig. 4:**
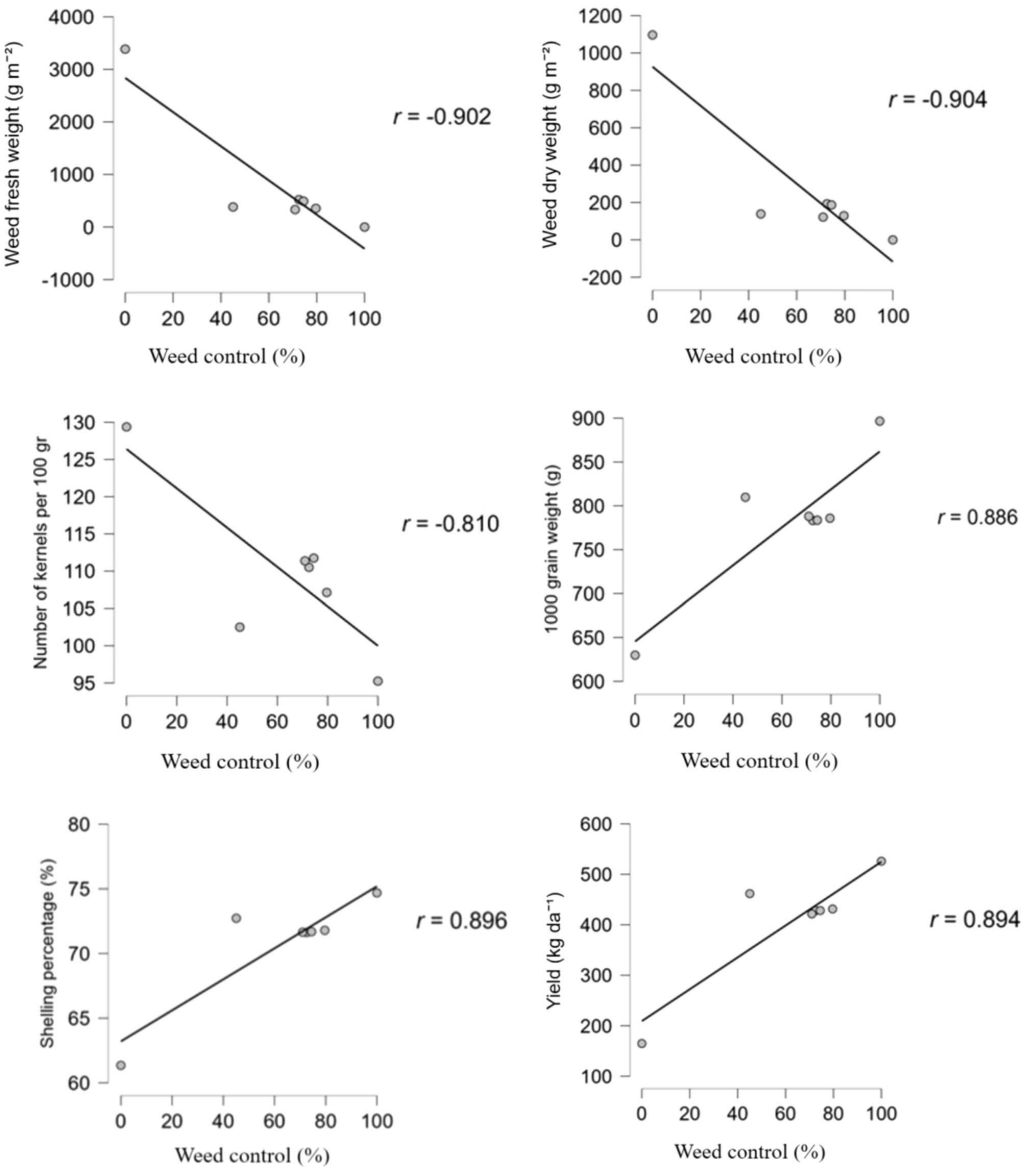
Correlation between the weed control (%) of herbicides on weed density and weed fresh, dry weight and peanut yield components.

A negative relationship was found between the weed control (%) of herbicides on the weed density and the fresh and dry weights of weeds. Fresh weight data were included only to support dry weight trends and were not used as a primary response variable for herbicide efficacy. A strong negative correlation was found between the weed control (%) rate and the fresh weight (-0.902) and dry weight (-0.904) of weeds. This relationship was statistically significant (p < 0.01) and it was observed that as the weed control (%) ratio increased, the fresh and dry weights of weeds decreased. In addition, it was determined that there was a negative correlation between the weed control (%) applied to weeds and the 100 g kernel count of peanuts and a positive correlation with other yield elements. A statistically positive correlation was found between the weed control (%) ratio and peanut yield (r = 0.894, p = 0.007) at the 1% level (Fig. 4). These strong positive correlations show that effective control of weeds by herbicide applications provides an improving effect on peanut production parameters. In cases where the weed control (%) increases, the fresh and dry weights of weeds decrease and this situation is positively reflected in peanut production parameters. Therefore, a high effect percentage is an important factor in increasing peanut yield and quality.

**Fig. 5:**
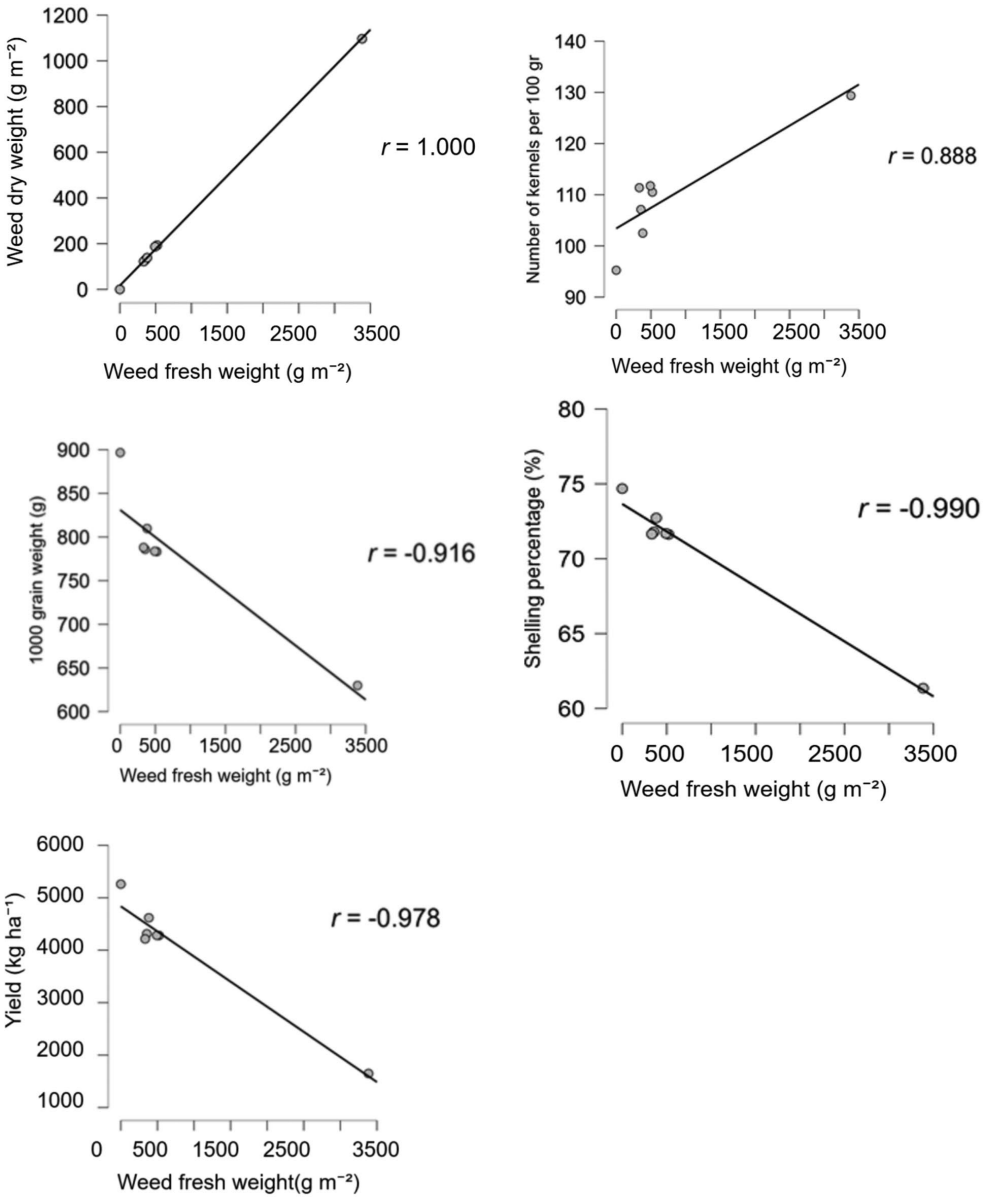
Correlation between weed fresh weight and peanut yield components.

Since r = 1.000 and p < 0.001 are between the fresh and dry weights of weeds, the relationship between these two parameters is completely positive and only the fresh weight of weeds was taken into account in the correlation analysis with peanut yield parameters. A very strong negative relationship (-0.978) was found between the fresh weight of weeds and peanut yield. This result shows that the presence of weeds has a significant negative effect on peanut yield and that herbicide applications are effective in increasing yield. This relationship is statistically significant (p < 0.001) (Fig. 5). These results demonstrate the strong negative impact of weeds on peanut production. Effective weed control increases yield components and overall peanut yield, highlighting the critical role of herbicides as a key management tool in peanut cultivation.

**Fig. 6:**
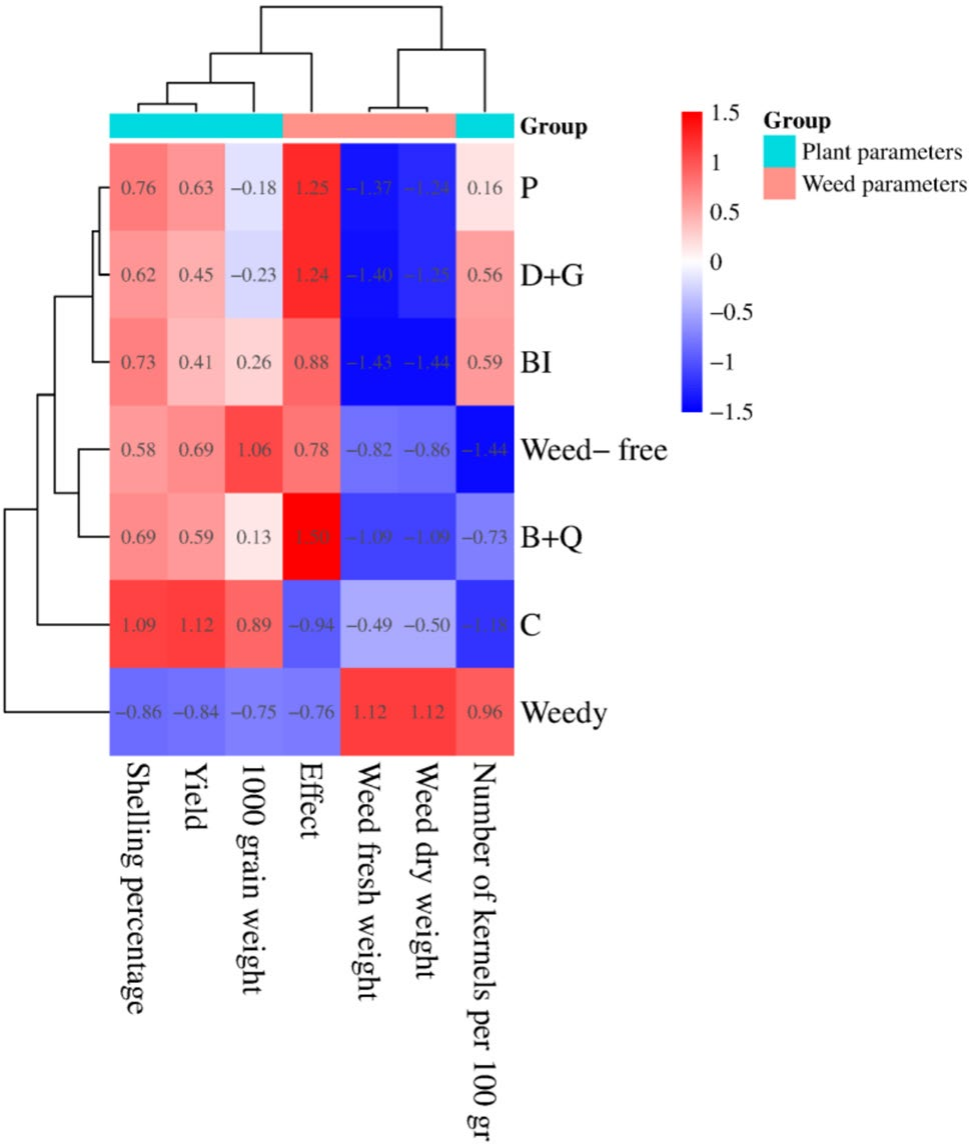
Heat map clustering of treatments and parameters. D: Dimethenamid-P, P: Pendimethalin, C: Clethodim, BI: Bentazon + Imazamox, G: Glyphosate isopropylamine salt, Q: Quizalofop-p-ethyl, and B: Bentazon.

The heat map clustering revealed two clearly separated main groups (Fig. 6). The first main cluster included only the weedy control plot. The second main cluster was divided into two subclusters; where clethodim herbicide alone formed a group, bentazon + quizalofop-P-ethyl herbicide and weed-free control application formed a group together. The remaining herbicide treatments were grouped separately, indicating different system-level response patterns among the herbicide programmes.

**Fig. 7:**
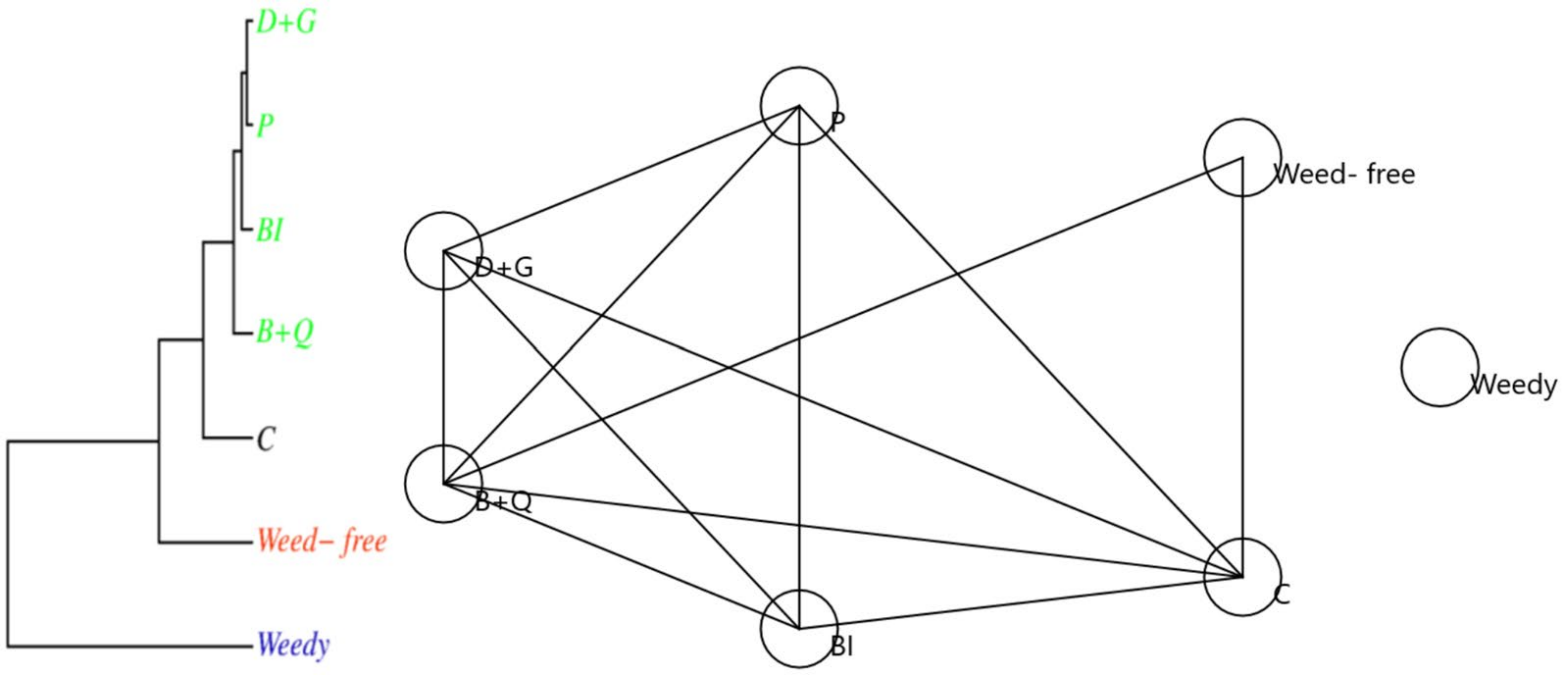
Hierarchical clustering and network graph analysis of applications. D: Dimethenamid-P, P: Pendimethalin, C: Clethodim, BI: Bentazon + Imazamox, G: Glyphosate isopropylamine salt, Q: Quizalofop-p-ethyl, and B: Bentazon.

The hierarchical clustering network analysis revealed a clearstructural seperation among treatments (Fig. 7). The weedy control group was completely isolated, while the weed-free control group was distinct from the herbicide treatment groups, indicating different system-level responses to the various management strategies. Multivariate analyses provided holistic and complementary information beyond traditional ANOVA approaches by revealing functional similarities and differences between herbicide programs. Heat map and hierarchical clustering analyses (Figs. 6 and 7) showed that the weed control group consistently formed an isolated cluster, clearly confirming that untreated plots were structurally separate from all herbicide-based management strategies. More importantly, the close clustering of the bentazone + quizalofop-P-ethyl combination with the weed-free control group revealed that this treatment not only reduced weed density and biomass but also generated a system-level response similar to mechanical weed control. This type of modeling approach is rarely clearly demonstrated in peanut weed management studies, where treatment effects are mostly reported through univariate comparisons.

The distinctly separate subcluster formation of the Klethodim application reflects its narrow-spectrum selective effect in suppressing turf weeds but not adequately controlling broadleaf species, thus creating a unique multivariate response profile. Similar partial-spectrum behaviors of ACCase inhibitors have been previously reported in peanuts and other summer crops^22,36^, however, this study clearly demonstrates how this selectivity translates into a distinctive clustering pattern when weed density, biomass, and yield parameters are evaluated simultaneously.

These findings highlight the need to evaluate herbicide programs not only based on short-term weed suppression levels but also on their holistic effects on weed communities and crop yield. The clustering structures and network relationships observed in this study demonstrate that herbicide mixtures capable of targeting both monocotyledonous and dicotyledonous weeds produce system-level results closer to weed-free conditions. This approach offers a novel, data-driven framework for comparing herbicide strategies and strongly supports the use of multivariate methods in guiding integrated weed management decisions in peanut production.

**Fig. 8:**
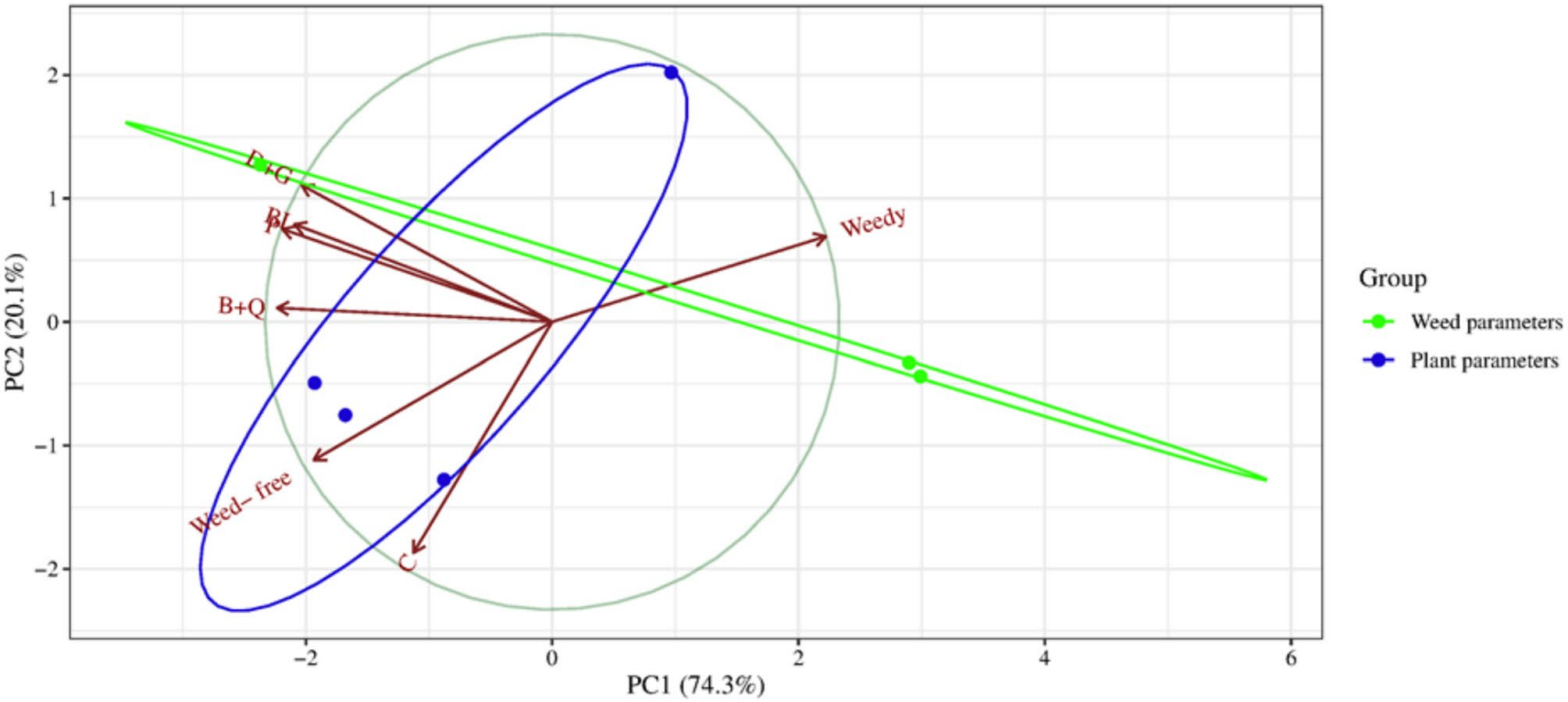
Principal component analysis of parameters and applications. D: Dimethenamid-P, P: Pendimethalin, C: Clethodim, BI: Bentazon + Imazamox, G: Glyphosate isopropylamine salt, Q: Quizalofop-p-ethyl, and B: Bentazon.

In order to better understand the effects of different herbicide applications on peanut yield and other yield elements, and to determine which factors have the greatest effect on yield by evaluating the effects of variables together, principal component analysis was performed. Peanut parameters and weed control (%), weed fresh and dry weight were distributed on a biplot pair (Fig. 8). Accordingly, the first two components (PC1: 74.3% and PC2: 20.1%) explained 94.4% of the variability of the original data. Such a high explained variance clearly shows that principal component analysis can be successfully used to evaluate the effect of the estimated parameters together with the applications. The first component (PC1) is negatively correlated with the weedy control (with -5.52 points), while it is positively correlated with the other applications (Fig. 8).

Advanced analyses such as correlation, heat map clustering, hierarchical clustering, network drawing analysis and principal component analysis, which we conducted on the mean values of the variables in the study, support the variance analysis, and we revealed the differences between the applications by clustering and correlating the findings. In general, the effects and relationships between the applications and parameters are clearly stated.

## Conclusions

In this study, the relationship between the effects of herbicides on weed density and peanut yield was examined comprehensively and notable findings were obtained. Annual changes in weed density and herbicide performance were carefully assessed, and herbicide applications showed differential effects on weed density and species composition. Herbicide effectiveness increased notably at 21 and 35 DAT and partially declined by 105 DAT. Bentazon + quizalofop-P-ethyl and bentazon + imazamox were highly effective in reducing weed fresh and dry biomass; however, bentazon + quizalofop-P-ethyl provided the highest protection against peanut yield loss. Herbicide efficiency showed strong negative correlations with weed fresh and dry weights, indicating that improved weed suppression was closely associated with yield protection. Herbicide treatments that effectively suppressed weeds substantially prevented yield loss relative to the weedy control, with yield protection averaging 60–70%. Overall, these results demonstrate that minimizing weed competition is essential for maintaining peanut yield potential. The use of high-efficacy herbicide combinations, together with appropriate application timing, plays a key role in successful weed management and in protecting peanut productivity.

## Data Availability

The raw data supporting the conclusions of this article will be made available by the authors on request.
